# Are life insurance futures a safe haven during COVID-19?

**DOI:** 10.1186/s40854-022-00411-z

**Published:** 2023-01-09

**Authors:** Kuan-Min Wang, Yuan-Ming Lee

**Affiliations:** 1 Department of Finance, Overseas Chinese University, 100 Chiao Kwang Road, Taichung, 40721 Taiwan; 2 Department of Finance, Southern Taiwan University of Science and Technology, No.1, Nantai St, Yung-Kang City, Tainan, Taiwan

**Keywords:** G11, G12, G22, Insurance futures, TVAR, TVP-VAR, Safe haven, Impulse response

## Abstract

This study aims to examine whether life insurance futures can serve as a hedge against the COVID-19 pandemic and whether they have the characteristics of a safe haven under the impact of the health shocks of the COVID-19 pandemic. We chose three life insurance stock futures in India and one in Taiwan as samples, including the market index of the two countries and the number of confirmed COVID-19 cases as sample variables. We used the growth rate of COVID-19 cases as the threshold variable, estimated the asymmetric threshold vector autoregression model, and found that insurance futures in the regime with a significant growth rate of confirmed COVID-19 cases can hedge against COVID-19 risks; therefore, insurance futures are a safe haven for the market. We further estimated the time-varying parameter vector autoregression model, and the impulse response results showed that insurance futures are a safe haven for COVID-19 pandemic risks.

## Introduction

The impact of health shocks on economic activities has received significant attention in recent years. Generally, when health shocks hit a family, there are two main economic costs: the medical cost incurred due to the disease that causes an increase in health expenditure and the decrease in labor supply and productivity, which causes a decline in income. Currently, most societies use insurance to finance these two types of costs. Therefore, when these two types of expenses increase, it may cause incomplete information in the insurance market, subsequently impacting the financial market. Traditional research on health shocks mainly concentrates on microeconomic analysis, such as the income of individuals and households, labor, health expenditures, and health insurance systems. However, empirical research on the impact of health shocks on the financial market is lacking.

Since December 2019, the COVID-19 pandemic has triggered a public health crisis. In addition to causing a large-scale loss of life, it also caused a significant financial crisis. Consequently, insurance coverage has become an essential topic for discussion—including individuals and enterprises facing high costs and losses, insurance coverage, whether insurance companies can hedge against the related risks, and whether insurance has the characteristics of a safe haven. Insurance is an essential financial instrument that can provide financial protection to people who have suffered economic losses due to disasters and diseases. As consumers and companies face economic challenges, the insurance industry and its customers continue to feel the effects of the COVID-19 pandemic. The pertinent financial and economic difficulties may affect the profitability of insurance companies in the future.

Regarding the impact of the insurance market on the economy, Skipper (2001) pointed out that the more developed the insurance market, the more outstanding its contribution to the economy. Insurance can maintain financial stability, promote effective allocation, and increase investment propensity. Haiss and Sümegi (2006) stated that the importance of insurance is growing gradually due to the increasing proportion of the insurance sector in the overall financial industry. Adams et al. (2009) pointed out that insurance is essential and critical in stimulating economic growth, especially in developing countries. Chau et al. (2013) stated that insurance could help alleviate unstable economic conditions. Vucetich et al. (2014) also pointed out that insurance can stabilize the economy, especially during a financial crisis. Grzegorz and Alina (2020a, b) discussed the impact of the COVID-19 pandemic on the insurance industry; according to them, when social risks increase, insurance has the effect of mitigating risks, which makes the public relatively optimistic.

How does the COVID-19 pandemic affect the insurance industry? Deloitte reported in 2020 that the insurance industry is generally well prepared for significant loss events, including pandemics. Still, the financial impacts will take time and be (re)insurer-specific. Insurers are responding to the widening COVID-19 outbreak on multiple fronts–claims payers, employers, and investment managers. Each of these has distinct challenges for the insurance industry, the global economy, and society. The financial impact of COVID-19 on (re)insurers depends on the circumstances of each enterprise– the classes and mix of businesses they underwrite, their pricing and reserving, policy wording, and reinsurance coverages. Falling equity markets and interest rates could pressure (re)insurers’ balance sheets, life insurance product profitability, and investment management fees related to savings products. There will be some time lag in insurance claims being notified to insurers, assessed, and finally, paid. Insurers have commenced evaluating their claims reserves considering the current circumstances, and this is expected to continue as fact patterns emerge. (Re)insurers with well-diversified risk portfolios will be the most insulated from the losses arising from the COVID-19 pandemic. Conversely, those with a high concentration of classes of business most exposed to the pandemic could be adversely impacted.

Balasubramanian et al. (2020) stated that the COVID-19 pandemic has reduced society's standard of living and lowered interest rates, leading to higher insurance premiums and more cautious underwriting methods for policyholders, which has reduced the demand for life insurance. Therefore, more emphasis may be placed on personal risk underwriting. Additionally, insurance companies may apply grace periods more frequently when signing insurance contracts for international travel, especially to countries with a high number of confirmed COVID-19 cases. Conversely, the COVID-19 pandemic may accelerate health insurance sales, daily hospitalization benefits, and medical assistance. For example, Sandhu and Prasanna (2020) pointed out that after the SAS epidemic in 2003, China's medical insurance expenditures grew substantially. The same is true for Taiwan's anti-epidemic insurance during the COVID-19 pandemic.

On the other hand, the COVID-19 pandemic has brought about fundamental market drivers that are rare in the financial world, causing shocks in the stock, commodity, currency, and debt markets. Therefore, using futures to avoid the risks posed by the COVID-19 pandemic has become a top priority of savvy traders. Whether in response to stock market crashes or volatility in the commodity market, futures are a risk aversion indicator that is one step ahead of the pandemic outbreak. For example, Wu et al. (2005), Xu and Wan (2015), Banerjee et al. (2020), Banerjee (2021), and Bohl et al. (2011) found that in comparison with the commonly used spot market, the futures market can disseminate information to the price at a faster rate, thus facilitating effective price discovery and offering better hedges against market risks. Therefore, using futures to circumvent COVID-19-related fears is an intelligent move in the context of panic selling triggered by the pandemic.^1^

Life insurance aims to protect people's lives and health and helps alleviate the risks of public health shocks. Futures play the role of hedging, speculating, and discovering the price of commodities. The hedging function is the most important among these and is related to the financial markets. However, previous literature has not discussed the use of insurance futures issued by life insurance companies for their hedging function and safe haven characteristics during the COVID-19 pandemic. Suppose insurance companies can issue such financial instruments with hedging risks or become safe havens for investors’ funds. It will help insurance companies in strategic risk management and investors’ investment decision-making. This is the primary motivation for this study, which aims to fill this gap in the existing literature.

Recent literature discussing the hedging function and safe haven of financial commodities during the COVID-19 pandemic includes the following: Adekoya et al. (2021), Akhtaruzzaman et al. (2021), Salisu et al. (2021), Wang (2021), Wang and Lee (2021), and Chemkha et al. (2021) found that gold is a safe haven during COVID-19, Chemkha et al. (2021) found that Bitcoin is not suitable as a safe haven owing to its high price fluctuations. Arif et al. (2021) found that green bonds can be used as risk diversification assets for medium-and long-term equity investors. However, none of these studies state that the abovementioned financial instruments are a safe haven for directly avoiding the risk of COVID-19; additionally, none discuss the futures of insurance companies' stocks in this regard. The most significant difference between this study and the recent literature is that we examine the COVID-19 pandemic from the perspective of insurance and futures markets. We explore the following questions: are insurance futures a safe haven during COVID-19? If the answer to the preceding question is affirmative, does the effect of this safe haven vary with time? What is the impact of specific events on this safe haven?

The purpose of this study is to examine whether life insurance futures can hedge against the risks of COVID-19 and whether they have the characteristics of a safe haven. We first used the growth rate of the confirmed COVID-19 cases as the threshold variable and estimated the threshold vector self-regression (TVAR) model. Subsequently, we found that in the interval where the growth rate of the confirmed COVID-19 cases was greater than the threshold, futures can hedge against the risks of COVID-19. On the other hand, we tested whether insurance futures had a safe haven effect that changed over time and sustained event shocks, using the time-varying parameter autoregression (TVP-VAR) model. The estimation results showed that insurance futures are a safe haven for COVID-19 but that their effect is more effective in the short term (six days). The main contribution of this study is indicating to investors that futures issued by insurance companies are a financial instrument worthy of investment and specifying their function as a safe haven during the COVID-19 pandemic.

This study is divided into five sections: the first section presents the introduction, the second section includes the literature review, the third section explains the study methodology, the fourth section presents the empirical results, and finally, the fifth section presents the conclusion.

## Literature review

Early studies on health shocks include Smith (1999), who found that health shocks caused a reduction in American income and labor. Stephens (2001) found that health shocks caused a reduction in the long-term consumption of households. Levy (2002) found that American health shocks impacted the average income and that there was no significant difference between the insured and the uninsured. Lindelow and Wagstaff (2005) showed that health shocks reduced income and labor supply for household units and cash payments for health expenditures. However, it must be noted that people with health insurance were more protected during health shocks than those without health insurance. Therefore, insurance is often considered a safe haven for health shocks (Wang et al., 2018).

Regarding the impact on the insurance industry, Moorcraft (2020) states that the COVID-19 pandemic has been spreading worldwide. The expected economic consequences might be as severe as a 2% decrease in the global GDP. This implies that almost every sector, including insurance, is threatened. Chester et al. (2020) cite a McKinsey report that the insurance industry could lose USD 760 billion globally because of the pandemic. Grzegorz and Alina (2020a, b) classify potential negative consequences of the COVID-19 pandemic for the insurance industry based on desk research of current insurance industry reports and business news. Then, they categorize them into one of the five areas: insurance demand, insurance claims, operations of insurance carriers, social and customer relations, and insurance supervision and regulation. It is shown that the adverse effects of the COVID-19 pandemic on the insurance industry are severe and can be seen from many perspectives. Nevertheless, in the long run, one can expect an increase in social risk insight and a greater awareness of the role of insurance in mitigating the adverse effects of random incidents. However, this would be a relatively optimistic scenario.

Przybytniowski et al. (2022) identify the risk and likelihood of potential consequences of the COVID-19 pandemic on the business insurance market over medium- and long-term horizons. The possible implications of COVID-19 are outlined both about the insurance company client, for example, a change in the amount of the insurance premium under the insurance agreement, as well as concerning the insurer, for example, appearance of innovative and competitive offers Trott’s concept Special attention has been paid to how the insurer’s strategy (scenario analysis) may be used to build resilience to other crises as well as to the planning of emergency solutions. Actual events confirm the hypothesis that changes in the business insurance market dominate the losses in the aftermath of the pandemic.

During the set COVID-19 pandemic era, what kind of financial instrument is a safe haven? Salisu et al. (2021) tested the ability of gold to hedge against external shocks during the COVID-19 pandemic. They found that gold has been a safe haven during the pandemic period up till now, although it was more effective before the outbreak. Additionally, the empirical results further found that regardless of the period, the hedging ability of gold is better than that of U.S. stocks and other precious metals (e.g., silver, palladium, platinum, etc.). Hasan et al. (2021) tested the hedging effects of 12 assets on the U.S. stock market during the 2008 global financial crisis and the COVID-19 pandemic. They showed that silver and Islamic stock indexes were safe havens during the 2008 global financial crisis and that the Islamic stock index and Tether have been safe havens during the COVID-19 pandemic. Additionally, while gold and Bitcoin have hedging characteristics during severe market downturns, safe haven assets change over time.

Diniz-Maganini et al. (2021) examined the price efficiency and correlation between Bitcoin, gold, the U.S. dollar index, and the Morgan Stanley Capital International World Index (MSCI World) during the COVID-19 period. They found that gold can be considered a safe haven for investors holding the MSCI World Index and the USD Index. When the investment period exceeds 3 months, Bitcoin can be considered a safe haven for the MSCI World Index. Yarovaya et al. (2021) found that during the COVID-19 outbreak, Sukuk has the characteristics of a safe haven, and the spillover effect between the traditional stock market and the Islamic stock market has become more robust. Nonetheless, Bitcoin is not an essential determinant of these relationships.

Chemkha et al. (2021) used major world stock market indexes and currencies to conduct empirical analysis with a multivariate asymmetric dynamic conditional correlation (ADCC) model. They showed that Bitcoin and gold as safe haven assets could effectively reduce the risk of international investment portfolios. They also found that during the COVID-19 pandemic, gold is a weak safe haven, but Bitcoin is not a safe haven. Ji et al. (2020) used a continuous monitoring program to detect changes in the left quantiles of asset returns and evaluate whether it was possible to offset the tail changes in the stock index by introducing safe haven assets into a simple mean-square error portfolio; additionally, they calculated the cross-quantilogram between pairwise asset returns and compared the predictability of the direction of the left quantile during normal and COVID-19 periods. They found out that during the COVID-19 period, gold and soybean commodity futures could be used as safe haven assets. Finally, Raheem (2021) tested whether Bitcoin can hedge against uncertainties such as VIX, EPU, and oil shocks. Comparing before and after the occurrence of the COVID-19 pandemic, the empirical results showed that Bitcoin had a safe haven feature before COVID-19 but that this feature disappeared during the COVID-19 period.

Discussing the effect of spreading during the COVID-19 pandemic, Banerjee (2021) used the multivariate ADCC-EGARCH model to detect the financial spreading effect between China and its main trading partners during the pandemic; the empirical results showed that China has a significant financial contagion effect with most developed and emerging markets. Li et al. (2021) studied the impact of the COVID-19 pandemic on the G20 stock market. They found that during the COVID-19 crisis, the total volatility between the G20 stock markets increased significantly. The developed markets are the primary spillover communicators, and emerging markets are the leading spillover receivers. Jebabli et al. (2021) found that volatility transmission between markets during the COVID-19 pandemic exceeded the volatility during the 2008 global financial crisis. During the 2008 global financial crisis, the stock market was the net transmitter of energy market volatility. During the COVID-19 turmoil, there was evidence of asymmetric volatility spillover between the stock and energy markets.

Abuzayed et al. (2021) detected the effect of systemic risk spillovers between global and individual stock markets in the countries most affected during the COVID-19 pandemic. They used a tail dependence risk measurement method, including conditional risk values (CoVaR) and delta condition VaR (ΔCoVaR). In addition, they used a bivariate dynamic condition correlation (DCC) GARCH model for empirical analysis. The results showed that the bivariate systemic risk spreading effect between the global stock market and each stock market intensified with the spread of COVID-19. In addition, they observed more marginal extreme risks for transmission and reception between developed markets in Europe and North America than in Asian stock markets.

They are testing the safe haven properties. Rubbaniy et al. (2021b) use wavelet coherence analysis on the global COVID-19 fear index and soft commodities’ spot and futures prices to investigate the safe haven properties of soft commodities from 2020 to 2021. The findings show that each of the sampled soft commodities leads safe haven behavior in either the spot or futures markets and for the short- or long-term investors during COVID-19 times. In addition, the mean–variance portfolio analysis indicates that portfolios with commodity futures are less risky and more efficient than those with only stocks, thus robustly supporting the safe haven properties of soft commodities during COVID-19.

Han et al. (2016) use the cross-quantilogram to detect predictability from stock variance to excess stock return and investigate the systemic risk of individual financial institutions, such as JP Morgan Chase, Morgan Stanley, and AIG. Rubbaniy et al. (2021a) add to the inconclusive debate on the safe haven properties of cryptocurrencies during COVID-19 by analyzing the wavelet coherence framework on the global COVID-19 fear index, cryptocurrency implied volatility index and cryptocurrency returns. Analyzing a proxy shows that cryptocurrencies are safe haven assets and supports the view that long-term investors can invest in the cryptocurrency market to hedge risks during the COVID-19 pandemic. Finally, Rubbaniy et al. (2022) use the wavelet coherence framework on four major ESG stock indices from global and emerging stock markets and two proxies of the fear about COVID-19. They find a solid and positive co-movement between the health fear index of COVID-19 and returns on ESG stocks, suggesting the existence of safe haven properties in ESG stocks. However, they also observe a negative co-movement between the stock market-based proxy of COVID-19 and returns on ESG indices, suggesting that the safe-haven properties of ESG stocks are contingent upon the proxy of the COVID-19 pandemic.^2^

In some studies, the number of confirmed COVID-19 cases was directly added to the model for discussion. For example, Hashmi et al. (2021) used the quantile unit root and cointegration tests to integrate the characteristics of the number of COVID-19 cases, the number of deaths, and the stock price. The quantile–quantile regression (QQR) estimates the relationship between the dependent and independent variables. The cointegration test showed that the stock price had a long-run relationship with the number of confirmed COVID-19 cases; additionally, it showed that while the stock price had a weak positive correlation with the high-quantile stock price, it had a strong negative correlation with the low-quantile stock price. Rehman et al. (2021) studied the causal relationship between G7 stock returns and the number of confirmed COVID-19 cases using the wavelet coherence approach and found that the number of confirmed COVID-19 cases and the number of deaths both showed strong consistency with the G7 stock market; however, these variables showed a weak relationship with Canadian and Japanese stock markets.

Yu et al. (2021) constructed two pandemic anxiety indexes based on the volatility of reported cases and COVID-19 deaths to examine the dynamic linkage between these anxiety indexes and the stock markets of the BRICS and G7 countries. They found that the anxiety indexes fluctuated over time, but the overall trend declined—the correlation between stock market returns and the pandemic anxiety indexes changed over time. Furthermore, after the COVID-19 mRNA vaccine was announced, the correlation became weaker and fluctuated less. Similarly, in the present study, we directly applied the impact of COVID-19 in terms of the number of confirmed COVID-19 cases to the model.

## Methodology

### Threshold autoregression model (TAR model)

The Threshold autoregressive model (TAR) mainly distinguishes different systems through the "threshold variable" being greater than (less than or equal to) one or more threshold values Q. The threshold is resolute endogenously by the model and is not subjectively determined. The basic concept is to use grid search to find the optimal threshold value. When the sum square of error (SSR) reaches the minimum, then the optimal threshold value Q of the TAR model can be obtained. If the concept of TAR is introduced into the VAR model, it will become Threshold-VAR (after this referred to as TVAR). Assuming that the nonlinear threshold autoregression (TVAR) model of the 2-regimes is as follows:

1 $$Z_{t} = \left\{ \begin{gathered} (A^{1} + \sum\nolimits_{i = 1}^{L} {\varphi_{i}^{1} Z_{t - i} } + \sum\nolimits_{i = 1}^{L} {\phi_{i}^{1} dc_{t - i} )I(dc_{t - d} > Q) + \varepsilon_{t}^{1} } \hfill \\ (A^{2} + \sum\nolimits_{i = 1}^{L} {\varphi_{i}^{2} Z_{t - i} } + \sum\nolimits_{i = 1}^{L} {\phi_{i}^{2} dc_{t - i} )I(dc_{t - d} \le Q) + \varepsilon_{t}^{2} } \hfill \\ \end{gathered} \right.$$

$$Z_{t} = \left[ \begin{gathered} dp_{t} \hfill \\ dm_{t} \hfill \\ \end{gathered} \right],\;A^{k} = \left[ \begin{gathered} \alpha_{0}^{k} \hfill \\ \beta_{0}^{k} \hfill \\ \end{gathered} \right],\;\varphi_{i}^{k} = \left[ {\begin{array}{*{20}c} {\alpha_{1}^{k} } & {\alpha_{2}^{k} } \\ {\beta_{1}^{k} } & {\beta_{2}^{k} } \\ \end{array} } \right],\;\phi_{i}^{k} = \left[ \begin{gathered} \alpha_{3}^{k} \hfill \\ \beta_{3}^{k} \hfill \\ \end{gathered} \right],\;{\text{k}} = {1},{ 2},{\text{ i}} = {1},{ 2} \ldots ,{\text{L}}$$

$L$ is lag operator, $dc_{t - d}$ is threshold variable, *d* is lag-periods, $Q$ is the threshold value, ${\phi }_{1i},{\phi }_{2i}$ $A^{k}$, $\varphi_{i}^{k}$, $\phi_{i}^{k}$ are the parameters in the two regimes, $\varepsilon_{t}^{k} = \left( {\varepsilon_{\alpha t}^{k} \varepsilon_{\beta t}^{k} } \right)^{\prime}$ are the error term, E(${\varepsilon }_{t}$│Ω_t − 1_) = 0, E(${\varepsilon }_{t}^{2}$│Ω_t − 1_) = ${\sigma }^{2}$, and Ω_t-1_ is the information set of t − 1. I(.) is an indicator variable, $I(dc_{t - d} > Q)$ = 1, otherwise $I(dc_{t - d} \le Q)$ = 0. In addition, if there is a long-run equilibrium relationship between the variables, that is, a cointegration structure, an error correction term must be added to the model, so the TVAR model will be converted into a threshold vector error correction (TVECM) model as follows:

2 $$Z_{t} = \left\{ \begin{gathered} (A^{1} + \sum\nolimits_{i = 1}^{L} {\varphi_{i}^{1} Z_{t - i} } + \sum\nolimits_{i = 1}^{L} {\phi_{i}^{1} dc_{t - i} + \gamma_{i}^{1} e_{t - 1} )I(dc_{t - d} > Q) + \varepsilon_{t}^{1} } \hfill \\ (A^{2} + \sum\nolimits_{i = 1}^{L} {\varphi_{i}^{2} Z_{t - i} } + \sum\nolimits_{i = 1}^{L} {\phi_{i}^{2} dc_{t - i} + \gamma_{i}^{2} e_{t - 1} )I(dc_{t - d} \le Q) + \varepsilon_{t}^{2} } \hfill \\ \end{gathered} \right.$$

$e_{t - 1}$ is the error correction term and $\gamma_{i}^{k}$ is the error correction coefficient.

First, we use the Johansen (1988) cointegration test to examine a long-run equilibrium relationship between the futures price and the market index. The VAR or VECM model can be set correctly, avoiding the problem of ignoring essential variables in the model. In addition, according to the test of Tsay (1998), the null hypothesis is a linear VAR (VECM) model (no threshold effect), and the alternative hypothesis is a nonlinear VAR (VECM) model. The threshold variables are rearranged from small to large in the estimation process to construct an "arranged autoregression" (ARR). Tsay (1998) uses the recursive least squares method to obtain the predicted residuals of the sorted regression. Then, the standardized predicted residuals are used to construct the chi-square statistics to test whether there is a nonlinear feature. The threshold effect exists if this statistic can reject the linear null hypothesis.

In the two-regimes bivariate threshold model constructed in this paper, the optimal threshold is determined by the endogenization of the model, and through grid search from multiple estimation results, the residual sum of squares (RSS) as the basis for selecting the most suitable threshold.

### Time-varying parameter VAR model (TVP-VAR model)

We use the TVP-VAR model with stochastic volatility suggested by Primiceri(2005) and Nakajima (2011). The TVP-VAR model is constructed from the VAR model of Eq. (1) as:

3 $$Z_{t} = X_{t} \beta_{t} + A_{t}^{ - 1} \sum {_{t} \varepsilon_{t} }$$

The parameter $\beta_{t}$, ${\text{A}}_{t}$, and $\sum_{t}$ are all time-varying.

$$A_{t} = \left( {\begin{array}{*{20}c} 1 & 0 & \cdots & 0 \\ {\alpha_{2,1,t} } & \ddots & \ddots & \vdots \\ \vdots & \ddots & \ddots & 0 \\ {\alpha_{k,1,t} } & \cdots & {\alpha_{k,k - 1,t} } & 1 \\ \end{array} } \right)\sum {_{t} } = \left( {\begin{array}{*{20}c} {\sigma_{1,t} } & 0 & \cdots & 0 \\ 0 & {\sigma_{2,t} } & \ddots & \vdots \\ \vdots & \ddots & \ddots & 0 \\ 0 & \cdots & 0 & {\sigma_{k,t} } \\ \end{array} } \right)$$

$$X_{t} : = I_{s} \otimes (1,Z^{\prime}_{t - 1} , \ldots ,Z^{\prime}_{t - s} ).$$ $$a_{t} = (a_{21} ,a_{31} ,a_{32} ,a_{41} , \ldots ,a_{k} ,_{k - 1} )^{\prime}$$, $$h_{t} = \left( {h_{1t} , \ldots h_{kt} } \right)\prime$$ and $$h_{jt} =$$$$\log \sigma_{jt}^{2}$$, $$j = 1, \ldots ,k,t = s + 1, \ldots ,n.$$, Assuming that the parameters of Eq. (3) are random walks, and then

4 $$\begin{array}{*{20}c} \begin{gathered} \beta_{t + 1} = \beta_{t} + u_{\beta t} , \hfill \\ a_{t + 1} = a_{t} + u_{at} , \hfill \\ h_{t + 1} = h_{t} + u_{ht} , \hfill \\ \end{gathered} & {V = Var\left[ {\left( {\begin{array}{*{20}c} {\varepsilon_{t} } \\ {u_{\beta t} } \\ {u_{at} } \\ {u_{ht} } \\ \end{array} } \right)} \right]} & {\sim N\left( {0,\left( {\begin{array}{*{20}c} {I_{k} } & O & O & O \\ O & {\sum_{\beta } } & O & O \\ O & O & {\sum_{a} } & O \\ O & O & O & {\sum_{h} } \\ \end{array} } \right)} \right)} \\ \end{array} ,$$

$$t = s + 1, \ldots ,n,$$$$\beta_{s + 1} \sim N(\mu_{{\beta_{0} }} ,\sum_{{\beta_{0} }} ),a_{s + 1} \sim N(\mu_{{a_{0} }} ,\sum_{{a_{0} }} )\;{\text{and}}\;h_{s + 1} \sim N(\mu_{{h_{0} }} ,\sum_{{h_{0} }} ) \cdot \sum_{{h_{0} }}$$ is a diagonal matrix.

Nakajima (2011) proposes two kinds of impulse response function analysis that change with time: the forward prediction interval and fixed time points. Therefore, the purpose of constructing the TVP-VAR model is to conduct impulse response analysis, examine the impulse response of risk on futures returns and market returns, and determine whether stock future returns and market returns are safe havens for COVID-19. For the safe haven theory of insurance futures, we refer to the insurance futures price model construction theory of Cox and Schwebach (1992) and deduce the safe haven characteristics of insurance futures in the Appendix.

## Empirical results

The variables' names, codes, and research periods are listed in Table 1. The sample included three life insurance futures in India and one in Taiwan. The main research variables included the futures prices of insurance, the market stock price index, and the number of confirmed COVID-19 cases. The futures price data of the four insurance were obtained from the database of the financial website *Investing.com*; the stock price index data were obtained from the TEJ (Taiwan Economic Journal) database, and the number of confirmed COVID-19 cases in the two countries were obtained from the network database of the Center for Systems Science and Engineering at Johns Hopkins University in the U.S. In the empirical analysis; first, the natural logarithm of each variable was taken.^3^ In Table 1, *lp* represents the logarithmic futures price, *lm* is the logarithmic market stock index, and *lc* is the logarithmic confirmed number of COVID-19 cases. The first-order difference variable *dp* is the individual stock futures return, *dm* is the market return, and *dc* is the growth rate of confirmed COVID-19 cases. Table 1, the last column is the research period; we use daily data for empirical analysis.

**Table 1:** Variable codes, names and research period

Variable code	Futures price	Variable code	Market price	Variable code	Confirmed numbers of COVID-19	Research periods
*lp*_HDFC	HDFC Life Insurance Future price	*lm*_IND	India Mumbai Sensex 30 stock index	*lc*_IND	India COVID-19 Confirmed numbers	2020/03/02 ~ 2021/0714
*lp* _ICICI	ICICI Prudential Life Insurance Company Ltd Future price	*lm* _IND	India Mumbai Sensex 30 stock index	*lc*_IND	India COVID-19 Confirmed numbers	2020/05/02 ~ 2021/0714
*lp* _SBIL	SBI Life Insurance Future price	*lm* _IND	India Mumbai Sensex 30 stock index	*lc*_IND	India COVID-19 Confirmed numbers	2020/01/22 ~ 2021/0714
*lp* _TWN	China Life Insurance Future price	*lm* _TWN	Taiwan TWSE Capitalization Weighted Stock Index	*lc*_TWN	Taiwan COVID-19 Confirmed numbers	2020/01/30 ~ 2021/07/14

Data sources: Insurance futures price data from the Investing.com financial website database, the stock market index from the TEJ database, and the number of confirmed COVID-19 cases in the two countries from the Johns Hopkins University Center for Systems Science and Engineering database

We established four groups of models for empirical analysis. The variables combination of the three groups of models in India are as follows: the first one is {*lp*_HDFC, *lm*_IND, *lc*_IND} with a research period over 2020/03/02 ~ 2021/07/14; the second group consists of {*lp*_ICICI, *lm*_IND, *lc*_IND}, the research period being 2020/05/02 ~ 2021/07/14, and the third group comprises {*lp*_SBIL, *lm*_IND, *lc*_IND}, with a research period spanning 2020/01/22 ~ 2021/07/14. The variables combination in Taiwan is {*lp*_TWN, *lm*_TWN, *lc*_TWN}, and the research period is 2020/01/30 ~ 2021/07/14. The starting time of each group of models is based on the availability of data for the most extended period obtained from the database.

**Fig. 1:**
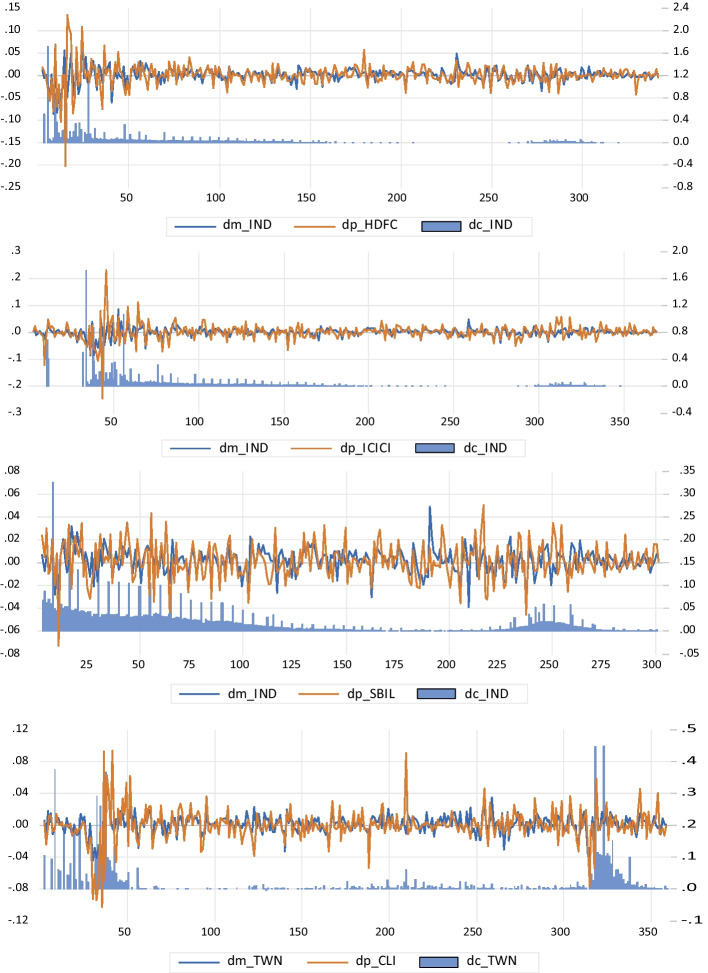
The trends of market return (*dm*), futures return (*dp*) and growth rate of COVID-19 (*dc*) in India and Taiwan (the left scale is the return rate, and the right scale is the confirmed growth rate)

The empirical analysis first determined whether the variables had stationary characteristics; therefore, we first performed a unit root test. Table 2 shows the Phillips–Perron unit-root test results for the variable-level and first-order difference terms. All the first-order difference term variables were found to be stationary, indicating that all variables had I(1) characteristics. To understand the time trend of the variables, Fig. 1 shows the trend of the first-order difference term of the variables. When the growth rate of confirmed COVID-19 cases fluctuated significantly, the returns of the futures and the market were observed to fluctuate wildly. However, fluctuations in the futures returns were substantially more significant than fluctuations in market returns. As seen from the volatility in Fig. 1, India and Taiwan both showed two more giant waves of COVID-19. Thus, when a significant wave of the disease occurred, futures returns and market system risks seemed to fluctuate significantly.

**Table 2:** Phillips Perron unit root test

Model	Constant	Constant + Trend	Constant	Constant + Trend
Variables	Level		First difference	
*lp*_HDFC	− 1.410	− 3.412**	− 20.155***	− 20.127***
*lp*_ICICI	− 1.330	− 3.118	− 19.609***	− 19.695***
*lp*_SBIL	− 1.554	− 3.077	− 18.593***	− 18.562***
*lp*_TWN	− 1.958	− 3.152*	− 19.415***	− 19.438***
*lm*_IND	− 1.585	− 2.069	− 19.718***	− 19.733***
*lm*_TWN	− 0.218	− 3.198	− 18.597***	− 18.586***
*lc*_IND	− 5.793***	− 3.115*	− 19.569***	− 20.430***
*lc*_TWN	− 1.944	− 2.503	− 16.298***	− 16.355***

The notation "***" and "**" represent 1% and 5% significance levels, respectively. The testing periods of *lp*_HDFC, *lp*_ICICI, and *lp*_SBIL is the research period indicated by the variables in the last column of Table 1. The testing periods of *lm*_IND and *lc*_IND is 2020/01/22 ~ 2021/0714, whichever is the longest period for test

Table 3 reports the descriptive statistics of the first-order difference term of the variables. Considering the mean, the futures return (*dp*) of SBIL is the highest among the four companies (0.0012); the stock market return (*dm*) in Taiwan (0.0012) is greater than that in India (0.0007), and the growth rate of confirmed COVID-19 cases in India (0.0476) is higher than that in Taiwan (0.0007). Regarding the standard deviation, the futures return risk is the highest for ICICI. India’s stock market risk (0.0163) exceeds Taiwan's (0.0128). Additionally, India’s growth rate risk of confirmed COVID-19 cases (0.1381) is greater than Taiwan's (0.0565). Overall, COVID-19 risks are greater than futures and stock market risks. The skewness coefficient shows that the futures and stock market are both left-skewed, whereas the growth rate of confirmed COVID-19 cases is right-skewed. The kurtosis coefficient shows that all variables are Leptokurtic. It can be seen that the futures and stock markets have continued downside risk, whereas the growth rate of confirmed COVID-19 cases has a continued upside risk. The Jarque–Bera (JB) value shows that all variables are not normally distributed.

**Table 3:** The descriptive statistics of the variables

Variables	*dp*_HDFC	*dp*_ICICI	*dp*_SBIL	*dp*_TWN	*dm*_IND	*dm*_TWN	*dc*_IND	*dc*_TWN
Mean	0.0006	0.0005	0.0012	0.0002	0.0007	0.0012	0.0476	0.0208
Std. Dev	0.0257	0.0310	0.0159	0.0210	0.0163	0.0128	0.1381	0.0565
Skewness	− 0.9420	− 0.4030	− 0.2646	− 0.2931	− 0.7503	− 0.3911	7.5797	4.8894
Kurtosis	18.376	22.574	4.4994	10.074	9.8553	7.0927	75.673	30.787
JB	3399.7	5789.1	31.605	749.49	742.83	258.26	83,127.5	12,908.1
P-value	0.0000	0.0000	0.0000	0.0000	0.0000	0.0000	0.0000	0.0000

JB is the value of Jarque Bera for normality testing. The testing periods of *lp*_HDFC, *lp*_ICICI, and *lp*_SBIL is the research period indicated by the variables in the last column of Table 1. The testing periods of *lm*_IND and *lc*_IND are 2020/01/22 ~ 2021/0714, whichever is the most extended period for the test

We further tested whether there was a long-run relationship between the four groups of model variables. We formulated the long-run relationship between futures prices, market index, and the number of confirmed COVID-19 cases as follows:

5 $$lp_{t} = b_{0} + b_{1} lm_{t} + b_{2} lc_{t} + e_{t}$$

The parameter $b_{1}$ measures the market system risk, also known as the risk aversion coefficient; $b_{2}$ measures the COVID-19 risk, that is, the COVID-19 risk aversion coefficient. When $b_{1}$ is greater than 0, the market system risk can be hedged, and when $b_{2}$ is greater than 0, the COVID-19 risk can be hedged. We conducted a cointegration test to measure whether the long-run relationship was stationary. Table 4 reports the results of Johansen's cointegration test. The Trace test andMax. Eigenvalue test results showed that only the Indian insurance company, HDFC Life Insurance, had a model that had a long-run cointegration relationship. According to the coefficients $b_{1}$ and $b_{2}$, it was observed that the four companies could hedge against market system risks, but that only HDFC Life Insurance could hedge against the risk of COVID-19. This reflected that the Indian company HDFC has long-term stability characteristics, while the other three do not have long-run hedging characteristics. Therefore, there is a need to test the short-run model's results further.

**Table 4:** Johansen cointegration test

Insurance company	Lags	H_0_	Trace test	Max. Eigenvalue test	$\varvec{b}_{1}$	$\varvec{b}_{2}$
HDFC	8	None	37.748***	28.257***	0.637***	0.007***
		At most 1	9.491***	9.491***		
ICICI	8	None	10.528	5.731	0.786***	− 0.003***
		At most 1	4.797**	4.797**		
SBIL	5	None	13.106	10.333	0.399***	0.005
		At most 1	2.773	2.773		
TWN	5	None	14.354	12.483	0.670***	− 0.033***
		At most 1	1.871	1.871		

The notation "***" and "**" represent 1% and 5% significance levels, respectively

As shown in Table 4, coefficient *b*_1_ of Eq. (5) is the risk aversion coefficient of the futures price relative to the stock market index, and *b*_2_ is the risk aversion coefficient of the futures price relative to the number of confirmed COVID-19 cases (this is the risk aversion elasticity of insurance futures facing the loss of a claim due to the occurrence of confirmed COVID-19 cases). HDFC alone has a cointegration relationship, which means that the risk aversion coefficient of HDFC has a mentioned value for investors. For example, the coefficient *b*_1_ = 0.637 implies that when the stock price index increases by 1%, the futures price increases by 0.637%, which can only hedge a 0.007% loss from the increase in the stock price index.

According to the cointegration test results and the short-term model's estimation, HDFC needs to estimate the VECM model owing to the cointegration relationship. The VECM model was formulated as follows:

6 $$\begin{gathered} dp_{t} = \alpha_{0} + \sum\nolimits_{i = 1}^{L} {\alpha_{1i} } dp_{t - i} + \sum\nolimits_{i = 1}^{L} {\alpha_{2i} } dm_{t - i} + \sum\nolimits_{i = 1}^{L} {\alpha_{3i} } dc_{t - i} + \gamma_{1} e_{t - 1} + \varepsilon_{\alpha t} \hfill \\ dm_{t} = \beta_{0} + \sum\nolimits_{i = 1}^{L} {\beta_{1i} } dp_{t - i} + \sum\nolimits_{i = 1}^{L} {\beta_{2i} } dm_{t - i} + \sum\nolimits_{i = 1}^{L} {\beta_{3i} } dc_{t - i} + \gamma_{2} e_{t - 1} + \varepsilon_{\beta t} \hfill \\ \end{gathered}$$

and the other three groups were composed as VAR models:

7 $$\begin{gathered} dp_{t} = \alpha_{0} + \sum\nolimits_{i = 1}^{L} {\alpha_{1i} } dp_{t - i} + \sum\nolimits_{i = 1}^{L} {\alpha_{2i} } dm_{t - i} + \sum\nolimits_{i = 1}^{L} {\alpha_{3i} } dc_{t - i} + \varepsilon_{\alpha t} \hfill \\ dm_{t} = \beta_{0} + \sum\nolimits_{i = 1}^{L} {\beta_{1i} } dp_{t - i} + \sum\nolimits_{i = 1}^{L} {\beta_{2i} } dm_{t - i} + \sum\nolimits_{i = 1}^{L} {\beta_{3i} } dc_{t - i} + \varepsilon_{\beta t} \hfill \\ \end{gathered}$$

Wald chi-square statistic was used to test the causality between two variables. When the null hypothesis $H_{0} :\alpha_{2i}^{{}} = ... = \alpha_{2L}^{{}} = 0$(that is, $H_{0} :dm\mathop{\longrightarrow}\limits^{ \times }dp$) is rejected, and $\sum\nolimits_{i = 1}^{L} {\alpha_{2i} }$ > 0, it is indicated that future returns can hedge against market system risks. When rejecting $H_{0} :\alpha_{3i}^{{}} = ... = \alpha_{3L}^{{}} = 0$ (that is, $H_{0} :dc\mathop{\longrightarrow}\limits^{ \times }dp$), and $\sum\nolimits_{i = 1}^{L} {\alpha_{3i} }$ > 0, it is indicated that futures returns can hedge against the COVID-19-related risks. When rejecting $H_{0} :\beta_{1i}^{{}} = ... = \beta_{1L}^{{}} = 0$($H_{0} :dp\mathop{\longrightarrow}\limits^{ \times }dm$), and $\sum\nolimits_{i = 1}^{L} {\beta_{1i} }$ > 0, it is indicated that market returns can hedge against the risk of future returns. When rejecting $H_{0} :\beta_{3i}^{{}} = ... = \beta_{3L}^{{}} = 0$ ($H_{0} :dc\mathop{\longrightarrow}\limits^{ \times }dm$), and $\sum\nolimits_{i = 1}^{L} {\beta_{3i} }$ > 0, it is indicated that market returns can hedge against the COVID-19-related risks.

After determining the causal relationship between the variables, we used the sum of the coefficients to judge whether the futures return can hedge against the market system risk and the risks of COVID-19 and whether market returns can hedge against COVID-19 risks. If the sum of the coefficients is positive, the risk can be hedged. Table 5 reports the linear model causality test results, according to Wald chi-square statistic, in which the futures and market returns of HDFC and ICICI show bidirectional causality. Conversely, TWN futures and market returns offer unidirectional causality. The $\sum\nolimits_{i = 1}^{L} {\alpha_{2i} }$ > 0 showed that the future returns of HDFC and ICICI can hedge against market system risks, and $\sum\nolimits_{i = 1}^{L} {\alpha_{3i} }$ > 0 showed that the futures returns of the two companies could hedge against the risks of COVID-19. However, $\sum\nolimits_{i = 1}^{L} {\beta_{3i} }$ < 0 showed that the market returns of the two companies could not hedge against the risks of COVID-19. Additionally, the sum of the coefficients of $\sum\nolimits_{i = 1}^{L} {\alpha_{3i} }$ and $\sum\nolimits_{i = 1}^{L} {\beta_{3i} }$ of TWN were positive, indicating that futures and market returns can hedge against the risk of COVID-19.

**Table 5:** The causality test in linear model

Insurance company	L	Model	${\varvec{H}}_{{\varvec{0}}} {\varvec{:dm}}\xrightarrow{{\varvec{ \times }}}{\varvec{dp}}$ $\left( {\sum\nolimits_{{{\varvec{i = 1}}}}^{{\varvec{L}}} {{\varvec{\alpha }}_{{{\varvec{2i}}}} } } \right)$	${\varvec{H}}_{{\varvec{0}}} {\varvec{:dc}}\xrightarrow{{\varvec{ \times }}}{\varvec{dp}}$ $\left( {\sum\nolimits_{{{\varvec{i = 1}}}}^{{\varvec{L}}} {{\varvec{\alpha }}_{{{\varvec{3i}}}} } } \right)$	${\varvec{H}}_{{\varvec{0}}} {\varvec{:dp}}\xrightarrow{{\varvec{ \times }}}{\varvec{dm}}$ $\left( {\sum\nolimits_{{{\varvec{i = 1}}}}^{{\varvec{L}}} {{\varvec{\beta }}_{{{\varvec{1i}}}} } } \right)$	${\varvec{H}}_{{\varvec{0}}} {\varvec{:dc}}\xrightarrow{{\varvec{ \times }}}{\varvec{dm}}$ $\left( {\sum\nolimits_{{{\varvec{i = 1}}}}^{{\varvec{L}}} {{\varvec{\beta }}_{{{\varvec{3i}}}} } } \right)$
HDFC	8	VECM	29.717*** (0.015)	38.523*** (0.603)	40.883*** (− 0.086)	67.129*** (− 0.071)
ICICI	8	VAR	44.082*** (0.555)	33.448*** (0.199)	23.129*** (− 0.024)	66.682*** (− 0.033)
SBIL	5	VAR	3.712 (0.110)	17.206*** (0.353)	5.138 (0.007)	5.947 (0.012)
TWN	5	VAR	8.942 (0.186)	14.660*** (0.364)	13.016** (0.048)	11.463** (0.032)

We use the Akaike information criterion (AIC) statistic to select the optimal Lag periods (L) and the Wald chi-square statistic to test the causality between variables. The notation "***" and "**" represent 1% and 5% significance levels, respectively; L represents the Lag periods

However, Jebabli et al. (2021), as mentioned in the preface, during the COVID-19 pandemic, volatility transmission between markets may have evidence of asymmetric volatility spillover. Therefore, we used the growth rate (*dc*) of confirmed COVID-19 cases as the threshold variable to perform a linear test to test the relationship between the returns of individual stock futures and the market returns and to determine whether there was an asymmetric effect and impulse response under the influence of the COVID-19 pandemic. Table 6 presents the results of the linear test. All four groups of models had significant threshold effects. Therefore, we formulated the asymmetric TVAR model as follows:

8 $${\text{Regime1}}\;\left\{ \begin{gathered} dp_{t} = \alpha_{0}^{1} + \sum\nolimits_{i = 1}^{L} {\alpha_{1i}^{1} } dp_{t - i} + \sum\nolimits_{i = 1}^{L} {\alpha_{2i}^{1} } dm_{t - i} + \sum\nolimits_{i = 1}^{L} {\alpha_{3i}^{1} } dc_{t - i} + \varepsilon_{\alpha t}^{1} \hfill \\ dm_{t} = \beta_{0}^{1} + \sum\nolimits_{i = 1}^{L} {\beta_{1i}^{1} } dp_{t - i} + \sum\nolimits_{i = 1}^{L} {\beta_{2i}^{1} } dm_{t - i} + \sum\nolimits_{i = 1}^{L} {\beta_{3i}^{1} } dc_{t - i} + \varepsilon_{\beta t}^{1} \hfill \\ \end{gathered} \right.\;dc_{t - d} > Q$$

9 $${\text{Regime2}}\left\{ \begin{gathered} dp_{t} = \alpha_{0}^{2} + \sum\nolimits_{i = 1}^{L} {\alpha_{1i}^{2} } dp_{t - i} + \sum\nolimits_{i = 1}^{L} {\alpha_{2i}^{2} } dm_{t - i} + \sum\nolimits_{i = 1}^{L} {\alpha_{3i}^{2} } dc_{t - i} + \varepsilon_{\alpha t}^{2} \hfill \\ dm_{t} = \beta_{0}^{2} + \sum\nolimits_{i = 1}^{L} {\beta_{1i}^{2} } dp_{t - i} + \sum\nolimits_{i = 1}^{L} {\beta_{2i}^{2} } dm_{t - i} + \sum\nolimits_{i = 1}^{L} {\beta_{3i}^{2} } dc_{t - i} + \varepsilon_{\beta t}^{2} \hfill \\ \end{gathered} \right.dc_{t - d} \le Q$$

**Table 6:** Linear test

Insurance company	Model	L	d	Chi-square statistic	Threshold value
HDFC	VECM	8	6	98.875***	8.161721%
ICICI	VAR	8	6	90.858***	0.131321%
SBIL	VAR	5	5	33.968**	0.243924%
TWN	VAR	5	1	70.683***	0.086630%

The notation "***" and "**" represent 1% and 5% significance levels, respectively; L represents the Lag periods, d represents the delay periods

The model residual square of sum (RSS) selected the most suitable threshold through grid search. The HDFC threshold was the largest among the four models at 8.16%, and the Taiwan threshold was the smallest at 0.08%. This means that all four short-term models had asymmetric volatility spillovers. Therefore, there may be errors in the estimation performed using only the linear model.

According to Table 6, there was a threshold for the four groups of models; therefore, short-term threshold model estimation was required. We set the interval for the growth rate of the confirmed COVID-19 cases to be greater than the threshold as in regime 1 and the interval for the growth rate of the confirmed COVID-19 cases to be less than or equal to the threshold as in regime 2. When estimating the bivariate threshold model, the confirmed growth rate was also added to the model in the form of exogenous variables, as shown in Eqs. (8) and (9), to test whether futures returns and market returns can hedge against the risks of COVID-19 and whether stock futures returns can hedge against market system risks.

Table 7 reports the estimated results of the threshold model. The causal relationship between the two variables was tested in each regime using Wald chi-square statistics. When rejecting the $H_{0} :\alpha_{2i}^{k} = ... = \alpha_{2L}^{k} = 0$(that is, $H_{0} :dm\mathop{\longrightarrow}\limits^{ \times }dp$), and $\sum\nolimits_{i = 1}^{L} {\alpha_{2i}^{k} }$ > 0, it is indicated that in the regime *k* (*k* = 1, 2), the futures returns can hedge against market system risk. When rejecting $H_{0} :\alpha_{3i}^{k} = ... = \alpha_{3L}^{k} = 0$ (that is, $H_{0} :dc\mathop{\longrightarrow}\limits^{ \times }dp$), and $\sum\nolimits_{i = 1}^{L} {\alpha_{3i}^{k} }$ > 0, it is indicated that future returns in the regime *k* can hedge against the risks of COVID-19. When rejecting $H_{0} :\beta_{1i}^{k} = ... = \beta_{1L}^{k} = 0$(that is, $H_{0} :dp\mathop{\longrightarrow}\limits^{ \times }dm$), and $\sum\nolimits_{i = 1}^{L} {\beta_{1i}^{k} }$ > 0, it is indicated that the future returns can hedge against the market system risk in regime k. When rejecting $H_{0} :\beta_{3i}^{k} = ... = \beta_{3L}^{k} = 0$ $\left( {H_{0} :dc\mathop{\longrightarrow}\limits^{ \times }dm} \right)$, and $\sum\nolimits_{i = 1}^{L} {\beta_{3i}^{k} }$ > 0, it is suggested that the market returns can hedge against the COVID-19 risk in regime *k*.

**Table 7:** The causality test in threshold model

Insurance company	Regime	Threshold model	${\varvec{H}}_{{\varvec{0}}} {\varvec{:dm}}\xrightarrow{{\varvec{ \times }}}{\varvec{dp}}$ $\left( {\sum\nolimits_{{{\varvec{i = 1}}}}^{{\varvec{L}}} {{\varvec{\alpha }}_{{{\varvec{2i}}}}^{{\varvec{k}}} } } \right)$	${\varvec{H}}_{{\varvec{0}}} {\varvec{:dc}}\xrightarrow{{\varvec{ \times }}}{\varvec{dp}}$ $\left( {\sum\nolimits_{{{\varvec{i = 1}}}}^{{\varvec{L}}} {{\varvec{\alpha }}_{{{\varvec{3i}}}}^{{\varvec{k}}} } } \right)$	${\varvec{H}}_{{\varvec{0}}} {\varvec{:dp}}\xrightarrow{{\varvec{ \times }}}{\varvec{dm}}$ $\left( {\sum\nolimits_{{{\varvec{i = 1}}}}^{{\varvec{L}}} {{\varvec{\beta }}_{{{\varvec{1i}}}}^{{\varvec{k}}} } } \right)$	${\varvec{H}}_{{\varvec{0}}} {\varvec{:dc}}\xrightarrow{{\varvec{ \times }}}{\varvec{dm}}$ $\left( {\sum\nolimits_{{{\varvec{i = 1}}}}^{{\varvec{L}}} {{\varvec{\beta }}_{{{\varvec{3i}}}}^{{\varvec{k}}} } } \right)$
HDFC		TVECM (L = 8, d = 6)				
	Regime 1	$dc_{t - 6} >$ 8.161721% s	94.280***	49.715***	29.819***	27.169***
			(0.932)	(1.290)	(0.101)	(0.032)
	Regime 2	$dc_{t - 6} \le$ 8.161721%	19.664***	9.102	38.933***	56.838***
			(− 0.208)	(0.247)	(− 0.165)	(− 0.069)
ICICI		TVAR (L = 8, d = 6)				
	Regime 1	$dc_{t - 6} >$ 0.13131321%	30.971***	30.703***	28.015***	34.459***
			(0.403)	(0.166)	(0.012)	(− 0.010)
	Regime 2	$dc_{t - 6} \le$ $0.131321$%	11.573	16.316**	20.688***	40.976***
			(− 0.377)	(− 1.990)	(5.051)	(4.079)
SBIL		TVAR (L = 5, d = 5)				
	Regime 1	$dc_{t - 5} >$ 0.243924%	3.481	15.162***	5.580	5.756
			(0.176)	(0.296)	(0.025)	(0.001)
	Regime 2	$dc_{t - 5} \le$ $0.243924$%	10.919**	16.662***	1.646	2.755
			(0.403)	(0.370)	(0.871)	(1.290)
TWN		TVAR (L = 5, d = 1)				
	Regime 1	$dc_{t - 1} >$ 0.086630%	10.631*	16.518***	12.304**	7.445
			(− 0.064)	(0.421)	(0.039)	(0.030)
	Regime 2	$dc_{t - 1} \le$ $0.086630$%	5.644	12.349**	8.949	12.313**
			(0.531)	(0.095)	(− 13.47)	(− 11.24)

We use Akaike information criterion (AIC) statistic to select the optimal Lag periods (L) and the Wald chi-square statistic to test the causality between variables. When the null hypothesis is rejected, there is a significant causal relationship between the variables. The notation "***", "**" and "*" represent 1%, 5% and 10% significance levels, respectively; L represents the Lag periods, d is delay periods. *TVECM* threshold vector error correction model, *TVAR* threshold vector autoregression model

The causality test and the positive value of $\sum\nolimits_{i = 1}^{L} {\alpha_{2i}^{1} }$ and $\sum\nolimits_{i = 1}^{L} {\beta_{1i}^{1} }$ showed that in regime 1, where the growth rate of confirmed COVID-19 cases is large, the futures returns of Indian, HDFC and ICICI, can hedge against the market system risks, while all the three companies can hedge against the risks of COVID-19; the sum value of $\sum\nolimits_{i = 1}^{L} {\alpha_{3i}^{1} }$ and $\sum\nolimits_{i = 1}^{L} {\beta_{3i}^{1} }$ showed that only HDFC can hedge against the risks of individual companies and the risks of COVID-19 simultaneously. However, the results were more inconsistent in regime 2, where the growth rate of confirmed COVID-19 cases was low. Only SBIL futures returns were found to hedge against market system risks; only SBIL and TWN futures returns were found to hedge against the risks of COVID-19, and only ICICI market returns were found to hedge against the risks of COVID-19. Therefore, according to Table 6, in regime 1, where the growth rate of confirmed COVID-19 cases was large, it can be concluded that insurance futures returns can hedge against the risks of COVID-19.

Hedge efficiency analysis. In Table 7, the values of $\sum\nolimits_{i = 1}^{L} {\alpha_{2i}^{k} }$ show the hedge ratio of futures return relative to the stock return, and $\sum\nolimits_{i = 1}^{L} {\alpha_{3i}^{k} }$ show the hedge ratio of futures return relative to the growth rate of confirmed COVID-19 cases. On comparing the values of $\sum\nolimits_{i = 1}^{L} {\alpha_{2i}^{k} }$ for the three models in India, the hedge ratio of HDFC (0.932) is the highest, and that of SBIL (0.176) is the lowest in regime 1, where the COVID-19 growth rate is greater than the threshold value. By contrast, in regime 2 where the COVID-19 growth rate is lower than the threshold value, the hedge ratio of SBIL (0.403) is the highest, and those of the other two models (HDFC and ICICI) are negative. If the coefficient sum $\sum\nolimits_{i = 1}^{L} {\alpha_{2i}^{k} }$ is positive, the higher the value, the greater the efficiency in hedging systematic risk.

In contrast, the Taiwan futures return hedge ratio is − 0.064 in regime 1 and 0.531 in regime 2. These indicate that when the COVID-19 growth rate is low (as in regime 2), Taiwan futures returns are more efficient in hedging systematic risk. As for the hedge ratio $\left( {\sum\nolimits_{i = 1}^{L} {\alpha_{3i}^{k} } } \right)$ of futures returns relative to the growth rate of confirmed COVID-19 cases, the hedge ratio of HDFC is 1.290 in regime 1 and is the highest, implying the efficiency in hedging the COVID-19 risks is the highest. In regime 2, however, SBIL shows a higher hedge efficiency. In the case of Taiwan, the hedging efficiency in regime 1 $\left( {\sum\nolimits_{i = 1}^{L} {\alpha_{3i}^{k} } = 0.{421}} \right)$ is higher than in regime 2 $\left( {\sum\nolimits_{i = 1}^{L} {\alpha_{3i}^{k} } = 0.0{95}} \right)$.

Our results have important implications for individual investors and insurance companies in suggesting particular insurance futures to enhance hedging efficiency and diversification of their portfolios. They can also assist policymakers in understanding and disentangling the health fear dimension among the various interlocking dynamics affecting insurance futures prices during the COVID-19 pandemic.

**Fig. 2:**
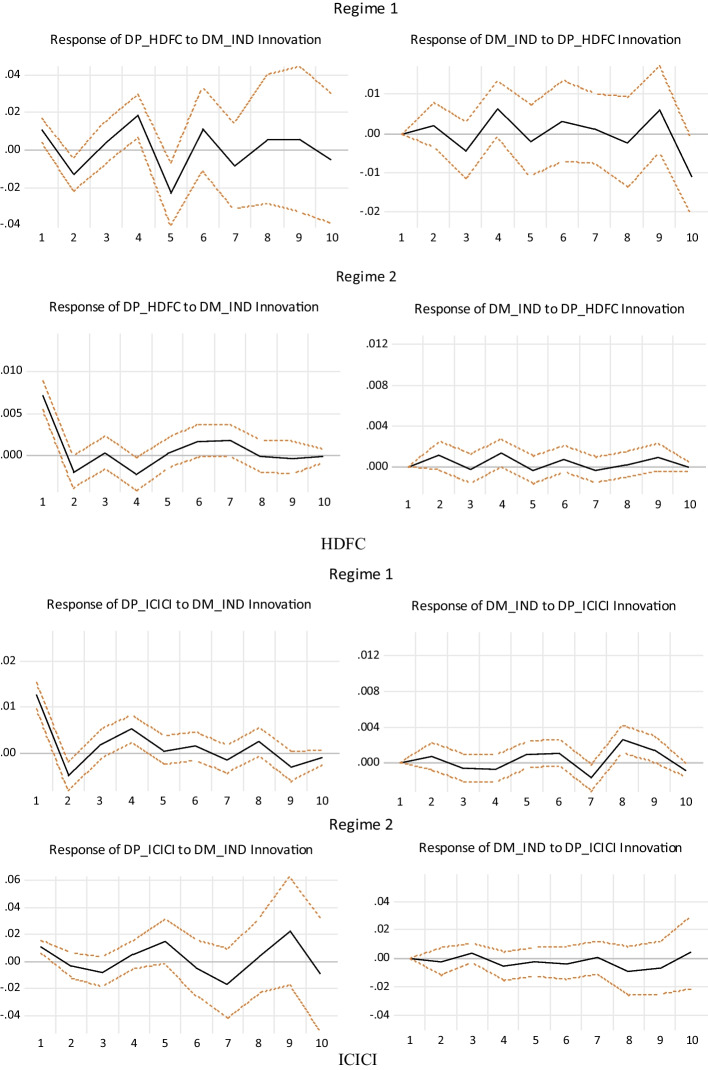

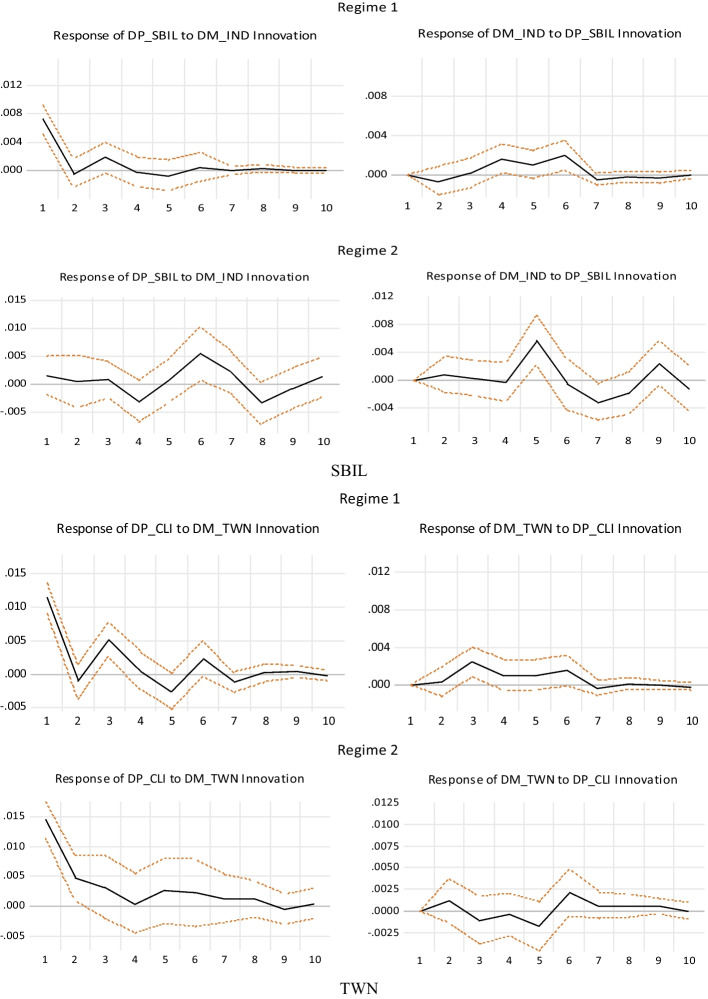
The impulse response analysis of asymmetric futures returns and market returns

To further understand the impulse response relationship between individual future returns and market returns under the asymmetric impulse responses, we conducted an asymmetric impulse response analysis based on the nonlinear impulse response function (NIRF), suggested by Koop et al. (1996), which is defined as follows:

10 $$NIRFy(k,t,\Omega_{t - 1} ) = E(Y_{t + k} |_{t} ,\Omega_{t - 1} ) - E(Y_{t + k} |\Omega_{t - 1} )$$

where $Y_{t + k}$ is the variable vector of period t + k, and Ωt − 1 is the t − 1 period impulse response information set. From Fig. 2, it is observed that the significant impulse response of futures returns to market system risks in regime 1 is greater than that in regime 2. The robust impulse response of futures returns (*dp*) to market (*dm*) system risks is greater than that of market system risks to futures returns. This means that in interval 1, where the risks of COVID-19 were high, the returns of individual futures can hedge against market system risks. Therefore, it can be concluded that during the COVID-19 pandemic era, insurance futures were a safe haven for market indexes.

**Fig. 3:**
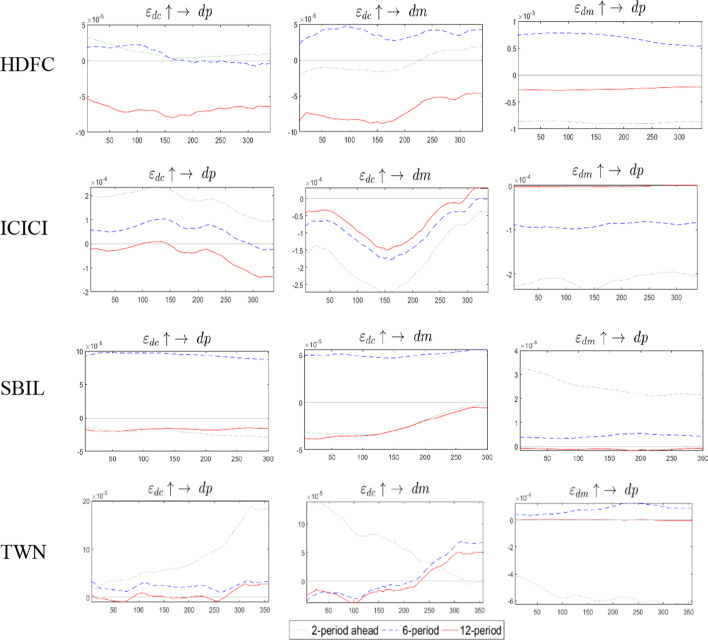
The 2th-period, 6th-period, and 12th-period out-of-forecasting impulse response analysis of TVP-VAR model shocks

We estimated the TVP-VAR model and conducted an impulse response analysis to understand the safe haven characteristics of insurance futures with time changes. Figure 3 reports the results of the TVP-VAR model out-of-sample forecasting the impulse response of the 2nd, 6th, and 12th periods, the $\varepsilon_{dc} \uparrow \to dp$ indicating the response of the future returns to the impulse of COVID-19 risks. The value of the response was found positive (or the effect was found to be positive), indicating that the futures returns can hedge against the COVID -19 risks; consequently, the futures returns are a safe haven for COVID-19. Similarly, a positive response value of $\varepsilon_{dc} \uparrow \to dm$ indicated that market returns are a safe haven for COVID-19, and a positive response value of $\varepsilon_{dm} \uparrow \to dp$ indicated that futures returns are a safe haven for market systemic risks.

According to the HDFC's impulse response results, regarding the impact of the COVID-19 risks on futures returns $\left( {\varepsilon_{dc} \uparrow \to dp} \right)$, a positive shock effect was observed in the 2nd and 6th periods, indicating that futures returns are a safe haven for COVID-19, but that their influence diminishes over time. The impact of the 12th-period is negative, meaning that there is no safe haven feature. From the effect of the COVID-19 risks on the market return $\left( {\varepsilon_{dc} \uparrow \to dm} \right)$, it is observed that only the 6^th^ period impulse has the characteristics of a safe haven. The above results indicated that in the short term (approximately one week), the safe haven characteristics of futures and market returns are more obvious, but this feature does not exist in the 12^th^ period (approximately two weeks). The results of ICICI show that only the 2nd and 6th periods of $\varepsilon_{dc} \uparrow \to dp$ are safe haven and that their effects decrease over time. The results of $\varepsilon_{dc} \uparrow \to dm$ showed that there is no safe haven for market returns, this indicates that market returns are not a safe haven for the COVID-19 risks. The $\varepsilon_{dc} \uparrow \to dp$ results of SBIL show that only the 6^th^ period futures returns have a safe haven feature; the $\varepsilon_{dc} \uparrow \to dm$ results show that only 6^th^ period market returns have a safe haven feature. Finally, the $\varepsilon_{dm} \uparrow \to dp$ results showed that 2nd and 6th futures returns are a safe haven for systemic risk, but that the effect of the 2nd period diminishes with time.

Regarding the results of TWN, the impacts of $\varepsilon_{dc} \uparrow \to dp$ are all positive shocks, and short-term shocks are better than long-term shocks (2nd period > 6th period > 12th period). Thus, Taiwan's futures have strong safe haven characteristics compared to Indian futures for the COVID-19 risks. Regarding the impact of the COVID-19 risks on the market returns $\left( {\varepsilon_{dc} \uparrow \to dm} \right)$, the safe haven characteristics of the 2nd period shock decrease over time, and the effects of the 6th and 12th periods gradually increase. Regarding the impact of market risk shocks on futures returns $\left( {\varepsilon_{dm} \uparrow \to dp} \right)$, only the 6th period shock has a positive effect; Thus, TWN futures is a safe haven for COVID-19 and the market risks. Based on the above results in India and Taiwan, it can be concluded that futures returns are a safe haven during COVID-19.

Figure 3 reports the impulse effects of short-term risk shocks. We used unexpected long-term risks and the TVP-VAR model to analyze the shock response at a specific time point for long-term risk shocks. First, we used Bai and Perron's (2003) structural change test and subsequently used the unexpected long-term error of Eq. (5) to detect the structural change points of unexpected risk. The results are shown in Table 8. There are two structural change points for HDFC and three structural change points for ICICI, SBL, and TWN.

**Table 8:** Bai and Perron (2003) structure change test

HDFC	2020-07-13	2020-11-11	
ICICI	2020-05-04	2020-08-05	2021-02-02
SBL	2020-07-10	2020-10-08	2021-01-20
TWN	2020-04-17	2020-07-24	2021-03-09

**Fig. 4:**
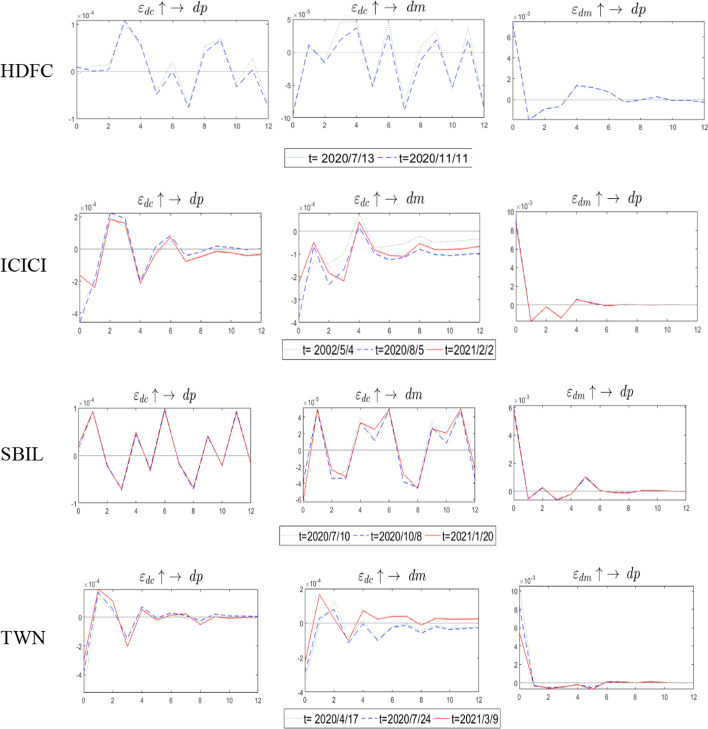
The Impulse response analysis of TVP-VAR model at specific structure change time

We used these change points to analyze the impulse response. In Fig. 4, the HDFC impact results show that comparing the impulse responses at two-time points, the impulse responses of $\varepsilon_{dc} \uparrow \to dp$,$\varepsilon_{dc} \uparrow \to dm$, and $\varepsilon_{dc} \uparrow \to dp$ are similar, and that there is a process of alternating positive and negative corrections. Comparing the impulse effects of $\varepsilon_{dc} \uparrow \to dp$ and $\varepsilon_{dc} \uparrow \to dm$, the duration of 2020/7/13 is greater than 2020/11/11. Comparing the corrected time, the recovery time of $\varepsilon_{dc} \uparrow \to dm$ is faster. While the impact results of ICICI are similar to those of HDFC, the difference is that there are more negative impact processes of $\varepsilon_{dc} \uparrow \to dm$. In general, 2020/05/04 was the most effective among the three-time points. The SBIL impact result is similar to that of HDFC, and the influence of $\varepsilon_{dc} \uparrow \to dm$ 2021/1/20 has a more significant effect. Compared with the outcome of India, Taiwan is faster and closer to zero in the positive and negative alternation correction range. The impact processes of the three times are similar, but the effects are of mutual magnitude.

The above results are summarized as follows: the $\varepsilon_{dc} \uparrow \to dp$ show that under the occurrence of unexpected risks, most of the positive effects show that futures are a safe haven for COVID-19 risks. Similarly, when a positive shock $\varepsilon_{dc} \uparrow \to dm$ exists, the market is a safe haven for COVID-19 risks. Finally, the result $\varepsilon_{dm} \uparrow \to dp$ shows that the safe-haven feature of futures for the systematic risk of the stock market is the strongest when the shock occurs, and then disappears quickly.

Insurance aims to protect personal health risks; this becomes especially relevant during the ongoing COVID-19 pandemic. The purpose of insurance companies issuing futures is to diversify portfolio risks and improve portfolio performance. The results of this study are more supportive within a large COVID-19 growth rate; insurance companies can hedge against the risk of COVID-19 and market systemic risk by issuing futures. Compared with Wu et al. (2005), Xu and Wan (2015), Banerjee et al. (2020), Banerjee (2021), and Bohl et al. (2011), this study has obtained further innovation. In addition to price discovery, insurance futures can also be a safe haven ahead of the outbreak of various COVID-19 variants. For investors, it is a financial instrument worthy of investment or utilized as a safe haven during COVID-19. This is the main contribution of the present study.

The recent discussion of safe haven literature (for example, Chemkha et al., 2021; Ji et al., 2020; Raheem, 2021, etc.) show more support for gold. Bitcoin is a safe haven for other financial indicators or commodities. Rubbaniy et al. (2021b) shows safe-haven behavior in the futures. The futures are issued by insurance companies, similar to the function of "reinsurance." In addition to the role of price discovery and reaction risk, we found that insurance futures have a safe haven function. In a regime with a significant growth rate of confirmed COVID-19 cases, insurance futures returns are a safe haven for COVID-19 risks and market system risks. Although the promotion of commodities such as "insurance + futures" are still in the stage of rapid development, the product structure tends to be standardized and innovative; given different varieties, different price trend analyses, and different risk preferences, insurance companies will have more autonomy. Consequently, the professional capabilities of future companies and the risk management function of the futures market will be better reflected.

## Conclusion

This study aims to test whether insurance futures can hedge against the risk of COVID-19 and possess the characteristics of a safe haven. We first consider the growth rate of confirmed COVID-19 cases as a threshold variable and estimate the TVAR model. According to our empirical results for long-term investment, only HDFC futures from among the futures of the three insurance companies in India present a systemic risk that is less than the market portfolio risk, have more substantial risk aversion characteristics, and can hedge the risk of COVID-19.

Concerning short-term investment strategies and the risk aversion efficiency of futures relative to the stock market, HDFC has the highest efficiency among the three insurance companies in India in Regime 1, which has a high COVID-19 growth rate. In Regime 2 where the COVID-19 growth rate is lower than the threshold value, SBIL has the highest risk aversion efficiency. The hedge ratios of the other two companies (HDFC and ICICI) are negative, indicating that the futures return and average return of the stock market move in opposite directions. As the average market return increases, the return on insurance futures decreases. For the risk aversion efficiency of futures relative to the stock market in Taiwan, both the negative hedge ratio in Regime 1 (− 0.064) and the positive hedge ratio in Regime 2 (0.531) indicate that the risk aversion efficiency of Taiwan China insurance futures is higher in Regime 2. This result stands in contrast with the Indian case. According to the risk aversion efficiency of futures returns relative to the COVID-19 growth rate in Regime 1, the hedge ratio of HDFC is 1.290, and the efficiency of the hedge against COVID-19 risks is the highest. In contrast, in Regime 2, SBIL has the highest hedge efficiency. As a result, Taiwan futures hedge efficiency is more elevated in regime 1 than in regime 2. The above results provide investors with important reference information when investing in futures and constitute one of the main contributions of this study.

Overall, our results show that insurance futures can hedge against the risks of COVID-19 in the regime where the growth rate of confirmed COVID-19 cases is greater than the threshold. Furthermore, from the analysis of the asymmetric impulse response between futures returns and market returns in Regime 1 with more significant COVID-19 risks, it is observed that futures returns can hedge against systemic market risks, indicating that insurance futures are a safe haven for market indexes. We further test whether futures have a safe haven effect that changes over time and when event shocks occur. The estimated results using the TVP-VAR model show that futures are a safe haven for COVID-19 risks and are more effective in the short term (6 days).

In the case of unexpected risks, the most positive effects show that insurance futures are also a safe haven for COVID-19 risks. Thus, the main implication of using TVAR and TVP-VAR in this study is that the safe haven characteristics of futures may vary with the magnitude of the growth rate of the confirmed COVID-19 cases or change over time. Therefore, we examine the impact of shocks in the case of change over time. Based on the findings of this study, we recommend relative risk diversification to the government and investors. This study provides necessary reference information that also forms the basis of our recommendation.

In future research, we aim to increase the number of variables observed (such as the national income), increase the number of sample countries, and conduct a comparative analysis of developed and developing countries.^4^ In terms of research methods, we aim to use wavelet coherence or cross-quantilogram models for analysis.

## Data Availability

The datasets are available from the corresponding author on reasonable request.

## Appendix

In this study, we refer to the insurance futures price model construction theory of Cox and Schwebach (1992) for the safe haven theory of insurance futures and deduce the safe haven characteristics of insurance futures.

First, we defer by making a specific assumption about the statistical nature of the aggregate claims while continuing the discussion in general terms. Let *S*(*t*) denote the aggregate claims reported (or paid) during the interval [0, *t*], the first *t* years of the policies in the market portfolio. Then, the final settlement value of the futures contract is

$$\left[ {S\left( T \right)/Q} \right] {1}00,000,$$

where Q is the aggregate premium paid on the market portfolio. Let *Y*(*t*) denote the market portfolio losses that are reported after the current time t but included in the settlement value:

$$Y\left( t \right) \, = S\left( T \right) \, - S\left( t \right).$$

Several statistical distributions are used in insurance claims process models to describe *Y*(*t*). At time t, the exchange announces the current loss ratio on the market portfolio. Rather than specifying a distribution for the claims process, at this point, we only assume that *Y*(*t*) has a finite mean and variance and that increments in *S*(*t*) (or log *S*(*t*)) are independent. These assumptions are commonly used in actuarial work.

Estimates of the distribution are made from the announced values of *S*(*u*) for various times *u *$\le$* t*. Estimates of these parameters depend on the distribution assumption for the claims process. For example, assuming a normal distribution, the representative sample mean and variance are the maximum likelihood estimators.

We denote the final settlement index by *F*(*T*). Then, dropping the 100,000 multiplier, we define it as:

$$F\left( T \right) = \frac{S\left( T \right)}{Q}$$

At times *t* < *T*, the futures price should be the expectation of F(T), conditional on the information available at time *t*. We assume that all market participants have the same information. This information includes all prior announcements about aggregate claims. Let *J*_*t*_ denote information available at time t. The information sets are assumed to be growing such that ${J}_{t}\supseteq {J}_{u}$ for $\mathrm{t }\ge \mathrm{ u}$, that is, we assume that ${J}_{t}$ includes the information generated by the loss process $S(u)$ for times $t \ge u$. This leads to the following formula for the futures price:

$$\begin{aligned} F\left( t \right) = & E\left[ {F\left( T \right){|}J_{t} } \right] \\ = & \frac{1}{Q}E\left[ {S\left( T \right){|}J_{t} } \right] \\ = & \frac{1}{Q}E\left[ {S\left( t \right) + Y\left( t \right){|}J_{t} } \right] \\ = & \frac{1}{Q}(S + E[Y\left( t \right)|J_{t} ] \\ \end{aligned}$$

where $S\left(t\right)=S$. Note that the index F(t) is always nonnegative.

Sherman (1991) discusses a similar method of pricing futures contracts. In this setting, the correct distributional assumption specifies that the logarithm of *S*(*T*)/*S*(*t*), conditional on *S*(*t*) = *S*, is normal with mean $\mu \tau$ and variance ${\upsigma }^{2}\uptau$, where $\tau =T-t$, and the futures price is

$$F\left( t \right) = \frac{1}{Q}\left\{ {S\left( t \right)E\left[ {\frac{S\left( T \right)}{{S\left( t \right)}}|J_{t} } \right]} \right\}$$

$$F\left( t \right) = \frac{S\left( t \right)}{Q}{\text{exp}}\left( {\mu \tau + \frac{{\sigma^{2} \tau }}{2}} \right)$$

Following the above equation, we take the logarithmic difference to get

$$\Delta \log F\left( t \right) = \Delta \log \left[ {\frac{S\left( t \right)}{Q}} \right] + \Delta \left( {\mu \tau + \frac{{\sigma^{2} \tau }}{2}} \right)$$

The above equation shows that future returns are affected by the mean and variance of total claims.

Baur and Lucey (2010, pp. 5–6) provide testable definitions of a hedge and safe haven: “a strict hedge is (strictly) negatively correlated with another asset or a portfolio on average,” and “a safe haven is an asset that is uncorrelated or negatively correlated with another asset or a portfolio in times of market stress or turmoil.” We think that COVID-19 is a type of market stress or turmoil for financial markets, which affects $\Delta \log \left[ {\frac{S\left( t \right)}{Q}} \right] + \Delta \left( {\mu \tau + \frac{{\sigma^{2} \tau }}{2}} \right)$. When insurance futures return $\Delta logF\left(t\right)$ can cover the loss of the mean of total claims *S*(*t*), it is expressed as $\mu \tau$ having a positive impact on Δ*logF*_*t*_ and indicates the characteristic of a hedge. Additionally, when the variance ${\sigma }^{2}\tau$ has a positive impact on Δ*logF*_*t*_, the insurance futures returns exhibit the feature of a safe haven.

We formulated the long-run relationship between futures prices, market index, and the number of confirmed COVID-19 cases as follows:

11 $$lp_{t} = b_{0} + b_{1} lm_{t} + b_{2} lc_{t} + e_{t}$$

The short-run model settings such as Eqs. (6) and (7), and (5)–(7) can be used to test the characteristics of a hedge, while impulse response analysis is used to test the feature of a safe haven.

## Abbreviations

COVID-19: Coronavirus disease 2019
TVAR: Threshold vector autoregression
TVP-VAR: Time-varying parameter vector autoregression
VIX: Volatility index
EPU: Economic policy uncertainty
ADCC-EGARCH: Asymmetric dynamic conditional correlation- exponential generalized autoregressive conditional heteroskedasticity
G20: Group of twenty
CoVaR: Conditional values at risk
ΔCoVaR: Delta condition conditional values at risk
QQR: Quantile–quantile regression
mRNA: Messenger RNA
SSR: Sum square of error
TVECM: Threshold vector error correction model

## Footnotes

1 For example, the use of futures to hedge against risks can help traders and investors to actively manage risks, such as shocks that may cause losses in the stock market: futures products provide a wealth of choices in the field of portfolio diversification. (For example, opening a short position in a micro E-mini or E-mini S&P 500, Nasdaq, or Dow Jones index futures contract that is delayed for 1 month greatly limits downside risks. In the event of a COVID-19-type shock resulting in stock market plunging, the gains from active short positions can be used to offset losses in traditional stock positions).

2 The wavelet coherence methodology has a large amount of computation. It is challenging to process it immediately; the selection of the mother wavelet is limited, and the transformation is too sensitive to the transfer the signal. When the input signal is shifted on the time axis, the corresponding wavelet conversion factors become elusive. Additionally, the cross-quantilogram is weak because it does not provide information about how the dependency varies with time. The method in this study can overcome these problems caused by the two methods. Apart from this, the two methods offer considerable advantages in discussing safe haven issues. The methodologies of wavelet coherence and cross-quantilogram can be used for future research and comparison.

3 During the research period of our study, India was one of the South Asian countries with relatively more confirmed cases, whereas Taiwan with fewer cases. Through this empirical comparison, we can understand the safe haven characteristics of insurance futures for two extreme cases. To this end, we select the insurance companies that issue futures in India and Taiwan from the Investing.com database as samples.

4 These are the current limitations of this study.
